# Motolimod, a selective TLR8 agonist induces apoptosis in monocytic myeloid-derived suppressor cells (M-MDSC)

**DOI:** 10.1186/2051-1426-3-S2-P296

**Published:** 2015-11-04

**Authors:** Zina J Rutnam, Yushe Dang, Gregory Dietsch, Hailing Lu, Yi Yang, Robert M Hershberg, Mary Disis

**Affiliations:** 1 UW School of Medicine Tumor Vaccine Group, Seattle, WA, USA; 2 VentiRx Pharmaceuticals, Seattle, WA, USA

## 

There is increasing evidence that myeloid-derived suppressor cells (MDSCs) contribute to the progression of cancer by inhibiting tumor-directed immune responses and producing mediators that promote the growth and survival of tumor cells. While present at low numbers in the peripheral blood of healthy individuals, levels of circulating MDSC increase in cancer patients as tumors produce factors that drive both the expansion and recruitment of these cells. MDSCs are a heterogeneous population and include both granulocytic MDSC (G-MDSC) and monocytic MDSC (M-MDSC) sub-populations, each of which can inhibit T cell function through arginase activity and the production of nitric oxide. Novel approaches to decrease MDSC expansion or inhibit their immunosuppressive functions hold great promise for augmenting tumor directed immune responses. In this study, we examined the effect of motolimod (formerly VTX-2337), a selective TLR8 agonist, on MDSC function. Initially TLR8 mRNA levels were assessed in MDSC isolated from the peripheral blood of healthy donors. TLR8 mRNA levels found in the M-MDSC subpopulation were comparable to those found in monocytes and myeloid dendritic cells (mDC), which express high levels of TLR8 protein and activate in response to motolimod. To further assess the M-MDSC response to TLR8 activation, PBMCs from healthy donors were treated with medium alone, low dose (167 nM), or high dose (500 nM) motolimod. Treatment with motolimod resulted in a significant, concentration–dependent loss of the M-MDSC (HLA-DR^-^CD14^+^) sub-population. In contrast, there was no significant difference in the recovery of the G-MDSC subtype (Lin-HLA-DR-CD33+) following motolimod treatment. Additionally, treatment with either the TLR7 agonist imiquimod, or TLR9 agonist CpG ODN2006, did not result in a loss of the M-MDSC population. The decrease in M-MDSC population was found to be the result of these cells undergoing apoptosis following motolimod treatment. M-MDSCs were also found to increase FAS expression in response to TLR8 activation by motolimod. Studies to determine if motolimod can decrease the immunosuppressive and/or tumor-promoting activities of MDSC sub-populations are ongoing. Our finding, that TLR8-activated m-MDSCs undergo apoptosis, suggests the potential for using motolimod to modulate MDSC numbers and function in cancer patients and enable a more effective, immune response to tumors. This innovative approach for modulating m-MDSC populations holds great promise for augmenting anti-tumor directed immune responses.

